# Association between particulate matter containing EPFRs and neutrophilic asthma through AhR and Th17

**DOI:** 10.1186/s12931-021-01867-w

**Published:** 2021-10-26

**Authors:** Jeffrey N. Harding, Maureen Gross, Vivek Patel, Steven Potter, Stephania A. Cormier

**Affiliations:** 1 Department of Biological Sciences, Louisiana State University and Pennington Biomedical Research Center, 6400 Perkins Rd, Baton Rouge, LA 70808 USA; 2 Division of Developmental Biology, Cincinnati Children’s Hospital Medical Center, University of Cincinnati College of Medicine, 3333 Burnet Avenue, Cincinnati, OH 45229 USA

**Keywords:** Combustion derived particulate matter, EPFRs, ScRNA sequencing, Aryl hydrocarbon receptor, Th17

## Abstract

**Background:**

Epidemiological data associate high levels of combustion-derived particulate matter (PM) with deleterious respiratory outcomes, but the mechanism underlying those outcomes remains elusive. It has been acknowledged by the World Health Organization that PM exposure contributes to more than 4.2 million all-cause mortalities worldwide each year. Current literature demonstrates that PM exacerbates respiratory diseases, impairs lung function, results in chronic respiratory illnesses, and is associated with increased mortality. The proposed mechanisms revolve around oxidative stress and inflammation promoting pulmonary physiological remodeling. However, our previous data found that PM is capable of inducing T helper cell 17 (Th17) immune responses via aryl hydrocarbon receptor (*Ahr*) activation, which was associated with neutrophilic invasion characteristic of steroid insensitive asthma.

**Methods:**

In the present study, we utilized a combination of microarray and single cell RNA sequencing data to analyze the immunological landscape in mouse lungs following acute exposure to combustion derived particulate matter.

**Results:**

We present data that suggest epithelial cells produce specific cytokines in the aryl hydrocarbon receptor (*Ahr*) pathway that inform dendritic cells to initiate the production of pathogenic T helper (eTh17) cells. Using single-cell RNA sequencing analysis, we observed that upon exposure epithelial cells acquire a transcriptomic profile indicative of increased *Il-17* signaling, *Ahr* activation, *Egfr* signaling, and T cell receptor and co-stimulatory signaling pathways. Epithelial cells further showed, *Ahr* activation is brought on by *Ahr*/ARNT nuclear translocation and activation of tyrosine kinase c-src, *Egfr*, and subsequently *Erk*1/2 pathways.

**Conclusions:**

Collectively, our data corroborates that PM initiates an eTh17 specific inflammatory response causing neutrophilic asthma through pathways in epithelial, dendritic, and T cells that promote eTh17 differentiation during initial PM exposure.

**Supplementary Information:**

The online version contains supplementary material available at 10.1186/s12931-021-01867-w.

## Background

Evidence links exposure to elevated levels of PM with deleterious health effects. Combustion-derived PM is generated by a variety of processes (e.g., burning of diesel/gasoline, stoves, cigarettes, etc.) and has been labeled a group 1 carcinogen by the World Health Organization in order to maintain and protect air quality and human health [21]. Particulate matter is generally categorized into three groups based on the diameter of the particles: course particulate matter, with a mean aerodynamic diameter < 10 µm, fine particulate matter, with an aerodynamic diameter < 2.5 µm; and ultra-fine particulate matter, with an aerodynamic diameter < 0.1 µm. While PM_2.5_ levels are generally below the national standard of 35 µg/m^3^ over a 24-h period, as advised by the U.S. Environmental Protection Agency (EPA), in major cities such as Los Angeles and in households, the levels can periodically exceed 150 µg/m^3^ [24, 61]. Even slight increases in PM_2.5_ levels show deleterious effects, as a study in 2013 demonstrated a correlation of more than 300,000 patients over nine countries and lung cancer frequency increasing by 36% per 10 µg/m^3^ increase [47]. It has been shown that organic pollutants chemically bond through transition metals, acting as intermediaries for environmentally persistent free radicals EPFRs [14, 15, 19, 31, 64]. The stabilized free radicals, as well as the transition metals present in particles, increase the production of reactive oxygen species (ROS) through Fenton chemistry, perpetuate the existence and stabilization of free radicals, and induce airway injury and inflammation [4, 22, 32, 34]. Increased ROS results in damage to tissues and disruption of cellular structure, inducing or exacerbating inflammatory responses. Many studies have demonstrated that acute exposure to elevated levels of PM elicits an inflammatory response within the lung and systemically causes oxidative stress. In human studies, it has been demonstrated that exposure to PM elicits increases in *Il-6*, *Gm-csf*, *Il-1β*, C-reactive protein, fibrinogen, and *Tnf*-α [62], as well as increases in pulmonary neutrophil numbers [45]. The activation of these pro-inflammatory cytokines and neutrophil invasion have been associated with increase in morbidity and mortality rates. Studies have also shown that increases of 10 µg/m^3^ in major cities corresponded to increases of up to 67% of all-cause mortality rates, as well as increased risk of atherosclerosis, immunological modifications, pulmonary oxidative stress, and a faster progression of chronic obstructive pulmonary disease (COPD) and cardiovascular diseases [40]. In addition, experiments with mouse models have shown PM activates NLRP3 inflammasome [30], promotes lung fibrosis [71], disturbs inflammatory cytokine homeostasis associated with changes in trace metal levels [39], compromises the antioxidant defense response [38], and increases the severity of respiratory infections [28, 49]. Therefore, the mechanism and response to PM is critical knowledge to fully understand PM exposure linked increase of morbidity and mortality.

In the current study, we determined the possible mechanistic pathways responsible for PM induced pathogenic T helper 17 (eTh17) response through epithelial activation of *Ahr* induced cytokines, dendritic cell cytokine activation of eTh17 specific cytokines, and gamma delta/natural killer T cell production of eTh17 specific cytokines. This is contrary to the activation of regulatory Th17 cells (rTh17) which have been shown to upregulate Ahr and IL-10 consequently allowing for overproduction of pro-inflammatory cytokines and neutrophil recruitment. We present data that suggest epithelial cells produce specific cytokines in the aryl hydrocarbon receptor (*Ahr*) pathway that inform dendritic cells to initiate the production of eTh17 cells.

## Materials and methods

### Particulate matter exposure

Both male and female C57BL/6J (Jackson) mice (aged 8–10 weeks) were used for the experiments. All mice were given free access to rodent chow and water and were maintained in a 12-h light-cycle environment. All animal protocols were written according to Policy for the Care and Use of Laboratory Animals and approved by the LSU Institutional Animal Care and Use Committee at Louisiana State University. We used a lab generated PM that contains EPFRs known as MCP230 (PM) with a mean aerodynamic diameter < 0.2 µm created and characterized by Dr. Lomnicki at Louisiana State University, as we have previously published [28]. Particles were suspended at a concentration of 1 mg/ml in sterile saline with 0.02% tween-80 PM particle solution was sonicated for 2 min at 30-s intervals on ice with a probe sonicator set to 50% amplitude. Mice were exposed to 50 µl of particle solution (vehicle) or 50 µl of PM for 4 h via oropharyngeal aspiration (OA), as previously described [27]. 50 µl OA exposure is based on the efficiency of instillation into the lungs and results in an inhalation exposure equivalent of 200 µg/m^3^ [53].

### RT2 PCR analysis

Following exposure, mice were euthanized, and their lungs were subjected to retrograde perfusion with 2 ml Hank’s Balanced Salt solution (HBSS) to remove red blood cells. We followed the manufactures protocol for RNA isolation and purification. We used the RT2 PCR kit for mouse drug metabolism (Catalog No. 330231) to analyze 84 genes related to the metabolism of PM particles in the lungs of (n = 10) mice. Analysis of the data was performed using the RT2 Profiler PCR Data Analysis tools on Qiagen’s website. We used the CT cutoff of 35 and the full panel geometric mean normalization method available in Qiagen’s analysis tool. Significance was calculated based on a Student’s t-test as p-value < 0.05.

### Single-cell dissociation of C57BL/6J mice

Following exposure, mice were euthanized, and their lungs were subjected to retrograde perfusion with 2 ml Hank’s Balanced Salt solution (HBSS) to remove red blood cells. The isolated lungs were dissociated using the gentle MACS Dissociator (Miltenyi Biotec) in 2 ml pre-warmed (37 °C) digest buffer (2 mg/ml Type 2 Collagenase, 1 mg/ml ProNase E, 62.5 U/ml DNAse 1, and 5 mM CaCl_2_ made up in DPBS without added calcium and magnesium) per 100 mg tissue. The lungs were further dissociated for 5 min using a 1000-µl pipette with an additional 1 ml pre-warmed digest buffer added. The lungs were then finely dissociated into a single-cell suspension using a ThermoMixer (Eppendorf) pre-warmed to 37 °C at 1200 RPM for 5 min. To remove clumps, the single cell suspension was passed through a 23-gauge needle and then filtered using 40 µM filter placed on top of a 50 ml conical tube and rinsed with 2 ml of 10% heat inactivated FBS/PBS solution. The resulting cell suspension was again filtered through a 40-µM filter to ensure that no clumping cells remained. Finally, the suspension was centrifuged at 1200*G* for 5 min, supernatant was removed, and cell pellet was suspended in 10 ml of 1% FBS/PBS solution. Barcoding of single cells was done using the Drop-seq protocol Version 3.1, by Dr. Steven Potter (Cincinnati Children’s Hospital), with a cell suspension of 100 cells/µl as previously described [46].

### Bioinformatics

After the droplets were sequenced on Illumina Nextseq500, we utilized the Drop-seq tools-2.3.0 pipeline to tag cell barcodes, tagged molecular barcodes, trimmed a 5ʹ primer sequence, trimmed a 3ʹ polyA sequence, converted the SAM filetype to Fastq, used STAR to align the sequences, sorted STAR alignment in queryname order, merged STAR alignment to M24 (GRCm38.p6) mouse genome, tagged SAM to recover cell/molecular barcodes, added gene/exon and other annotation tags, and conducted barcode repair. For downstream analysis, we removed cells with < 300 detected genes (transcript count > 0) and > 10% of transcript counts mapped to mitochondrial genes, which is indicative of broken cellular membranes, and removed genes with transcripts detected in < 3 cells in Seurat which is standard procedure (version 3.1.2;) [10, 41, 57]. After combining our vehicle and PM samples, this left us with 15,813 genes × 6118 cells and 15,851 genes × 5337 cells, respectively. The data were then processed using the sctransform normalization method with the standard Seurat data integration protocol. Clustering was performed with Seurat’s UMAP using significant principal components (PCs) determined by a JackStraw plot. PCs used to construct the UMAP were p < 0.05 and the elbow plot was used to determine the cutoff; the elbow and jackstraw plot are presented as Additional file 1. Marker genes differentiating each cluster were determined using Seurat’s FindAllMarkers function with the default settings. We used SingleR and the Immgen mouse genome database to classify our cell types by referencing the marker genes, derived from the FindAllMarkers function, with the Immgen database [1]. Differentially expressed genes were identified using the FindMarkers function in Seurat, which applied the Wilcoxon rank-sum test with a false discovery rate (FDR) < 0.05. The differential expression of each cell type was analyzed using R Clusterprofiler for gene ontology analysis, gene set enrichment analysis, and Kyoto Encyclopedia of Genes and Genomes (Kegg) pathway enrichment [70]. Genes in enriched pathways were individually identified to be activation factors or shown to increase signaling within the enriched pathway. The significance reported here was adjusted using Benjamini–Hochberg correction which controls the FDR and thus significance was determined to be FDR < 0.05 in the gene ontology analysis and gene enrichment analysis.

## Results

### RT2 profiler array of EPFR containing ultrafine particle exposure in vivo

We and others have observed PM affecting changes to epithelial cell integrity resulting in pulmonary neutrophilic infiltration, and metabolic dysregulation [2, 5, 33, 36, 40, 67]. However, the specific mechanisms by which PM causes these deleterious effects have yet to be discovered. To elucidate the effects that exposure of EPFR containing ultrafine particles (PM) has on the transcriptomic profile of mouse lungs, we exposed C57BL/6J adult mice to a laboratory generated particle with properties mimicking combustion derived PM but with defined chemical speciation (PM) and analyzed the lungs by the RT2 Mouse Drug Metabolism profiler PCR array. The PCR array showed 12 genes that were significantly increased following 4-h exposure to PM (Table 1). We saw significant increases in mRNA of Cyp1a1, Cyp4b1, Gpx2, Mt2, and Aldh1a1. Using pathway analysis in the Database for Annotation, Visualization and Integrated Discovery (DAVID) identified tryptophan metabolism, steroid hormone biosynthesis, retinol metabolism, glutathione metabolism, and thyroid hormone synthesis enrichment, suggesting that exposure to PM increases oxidative stress and elicits an antioxidant response.

**Table 1:** The RT2 Profiler array shows significant (p < 0.05) gene regulation of *Ahr* cytokines in PM compared to vehicle exposed (n = 5) mice after 4-h post exposure (n = 5)

Gene symbol	Name	Log_2_ fold change	p-value
Aoc1	Amine oxidase, copper-containing 1	1.63	0.001
Aldh1a1	Aldehyde dehydrogenase family 1, subfamily A1	1.44	0.006
Comt	Catechol-*O*-methyltransferase	1.68	0.040
Cyp1a1	Cytochrome P450, family 1, subfamily a, polypeptide 1	4.91	0.010
Cyp4b1	Cytochrome P450, family 4, subfamily b, polypeptide 1	1.48	0.011
Gpx2	Glutathione peroxidase 2	2.72	0.001
Gsr	Glutathione reductase	1.94	0.005
Hk2	Hexokinase 2	1.70	0.014
Mt2	Metallothionein 2	2.58	0.005
Nqo1	NAD(P)H dehydrogenase, quinone 1	1.69	0.004
Pon1	Paraoxonase 1	1.43	0.043
Pon3	Paraoxonase 3	1.26	0.039

### ScRNA-seq of in vivo exposure to PM pinpoints a distinct gene expression signature

**Fig. 1:**
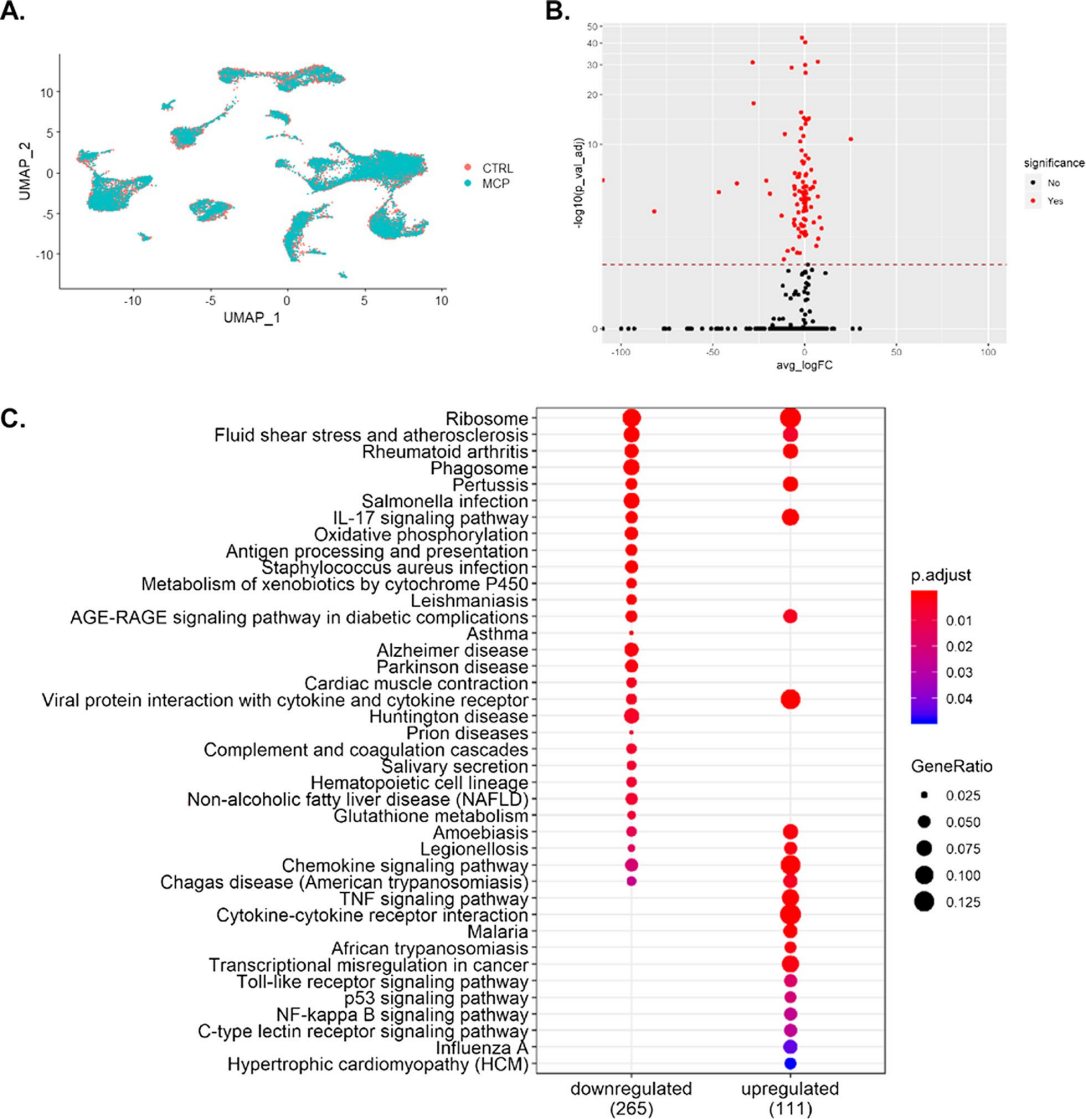
Exposure to PM induces transcriptomic changes across the entire lung at 4-h post exposure. **A** UMAP plot of all vehicle-exposed cells compared to PM-exposed cells showing a conserved cellular heterogeneity during 4-h timepoint. **B** Volcano plot showing fold change of 210 differentially expressed genes (DEG) over vehicle-exposed that were FDR < 0.05. **C** KEGG pathway analysis illustrating an increase in Il17 signaling, *Tnf* signaling, toll-like receptor signaling, and P 53 signaling

To determine from where the PM-induced expression of *Cyp1a1* originated, we performed scRNA-seq using the dropseq protocol established by the McCarrol Lab [41]. This allows us to understand the mRNA changes at a cellular level and explore each cell’s function, as the transcriptional program is a large determinant of the function. Following quality-control and pre-processing steps, selecting cells with at least three transcripts and 200 genes, we retained more than 6000 cells from our vehicle exposed and more than 5000 cells from our PM exposed groups (Fig. 1a). Comparing all RNA in PM-exposed to vehicle-exposed cells, over 18,000 distinct genes were detected overall, with 210 found to be differentially expressed (Fig. 1b). The analysis of the differentially expressed genes (DEGs) in the KEGG database indicated strong correlations of an upregulated *IL17* signaling pathway, viral protein interaction with cytokine and cytokine receptors, chemokine signaling, *Tnf* signaling, toll-like receptor signaling, and P53 signaling pathways (Fig. 1c). Interestingly, this suggests an increase in T-cell response; however, one of the downregulated pathways is antigen processing and presentation. Among the other downregulated pathways are pathways such as salmonella infection, phagosome, oxidative phosphorylation, and *Staphylococcus aureus* infection.

### In vivo exposure to PM evokes heterogenous responses from epithelial and dendritic cells

**Fig. 2:**
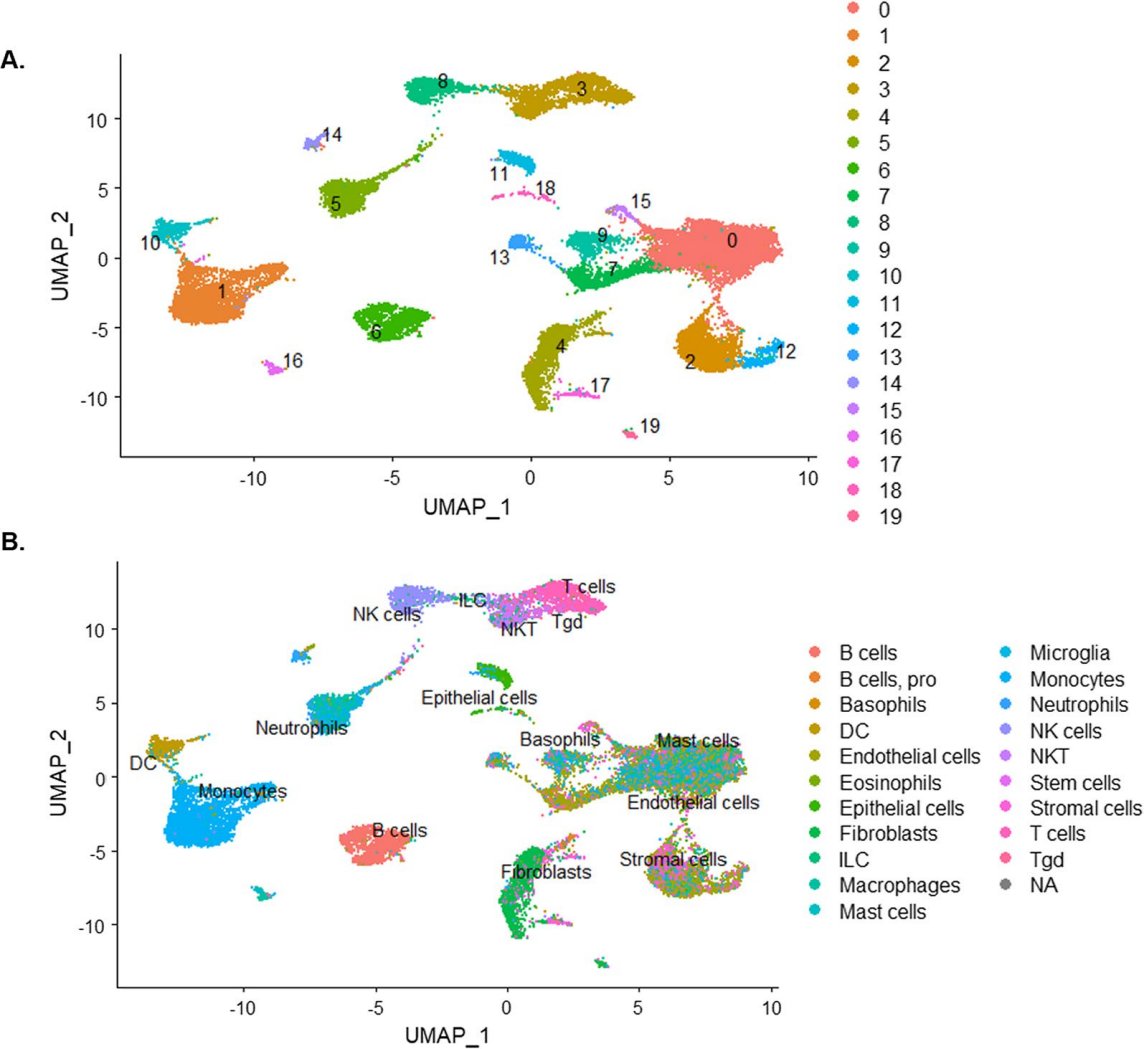
Single-cell RNA sequencing demonstrated 20 distinct cell clusters in PM-exposed mice lungs. **A** UMAP plot of the 20 distinct cell clusters. **B** UMAP plot labeled by single-cell passed through SingleR program for cell identification

**Fig. 3:**
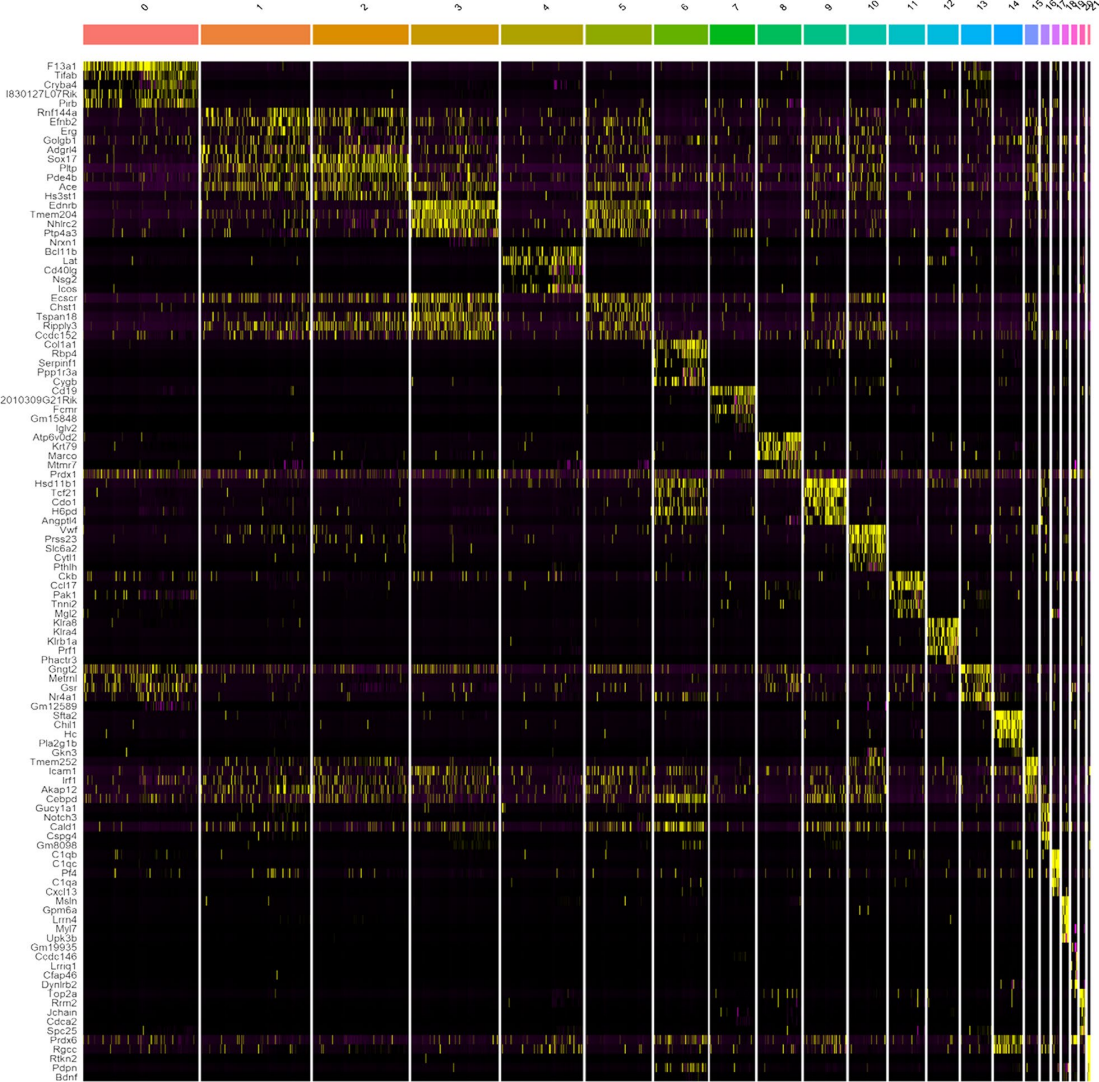
Heatmap of clusters from previous figure that show the top five genes that discriminate each individual cluster

**Fig. 4:**
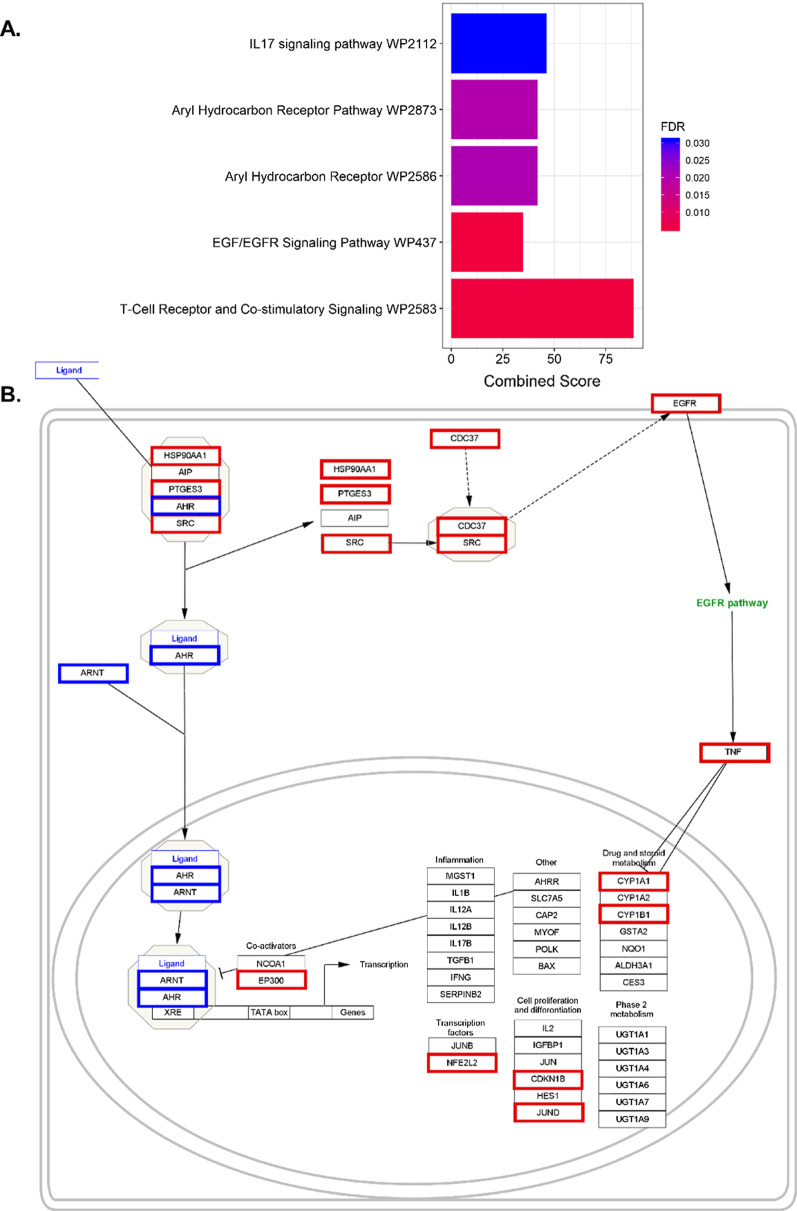
Enrichr pathway analysis showing **A** upregulated pathways in epithelial cells (characterized in Fig. 3) from lungs of mice exposed to PM compared to vehicle exposed. FDR (based on Bonferroni correction) < 0.05. **B** *Ahr* pathway that illustrates the upregulated genes in red and downregulated genes in blue showing an upregulation in *Egfr* and xenobiotic response elements Cyp1a1 and Cyp1b1. Combined score is a combination of the p-value and z-score (calculated by using a modification to Fisher’s exact test) calculated by multiplying the two scores

In comparing all mRNA of PM-exposed to vehicle-exposed cells in this study, we see significant changes to the function of epithelial cells and dendritic cell. To explore the underlying mechanisms, we focused on these specific cells. Utilizing Seurat [10, 57] and UMAP, we clustered the single cells into 20 distinct clusters (Fig. 2a). We found the top five differential gene expression profiles for each of the different identity clusters (Fig. 3) and passed those gene expression profiles through the Immgen database using the SingleR [1] to label the cells (Fig. 2b). This allows us to correctly identify the cell clusters in an unsupervised manner, while reducing biases and to evaluate the differential gene expressions across clusters of PM-exposed mice to vehicle-exposed mice. Through the use of Enrichr, we could ascertain that significant biological pathways were affected by short-term exposure to PM. The differential expression profile between vehicle- and PM-exposed epithelial cells was filtered for positively related gene expression and processed in Enrichr. From Enrichr, we saw five pathways directly related to the initiation of eTh17 cells. We saw increases in *Il-17* signaling, *Ahr* activation, *Egfr* signaling, and T cell receptor and co-stimulatory signaling pathways (Fig. 4a) in our epithelial cluster from Fig. 3. Here, we show that the transcription profile suggests an upregulation in cytokines that are activated via the *Ahr* pathway specifically in epithelial cells (Fig. 4b).

*Ahr* activation is known to induce c-src-dependent stimulation of *Egfr* and its downstream target *Erk*1/2 [20, 69]. This then leads to the upregulation of similar cytokines to *Ahr/Arnt* xenobiotic response elements like *Cyp1a1, Cyp1b1,* and *Cox-2* [20, 69].

**Fig. 5:**
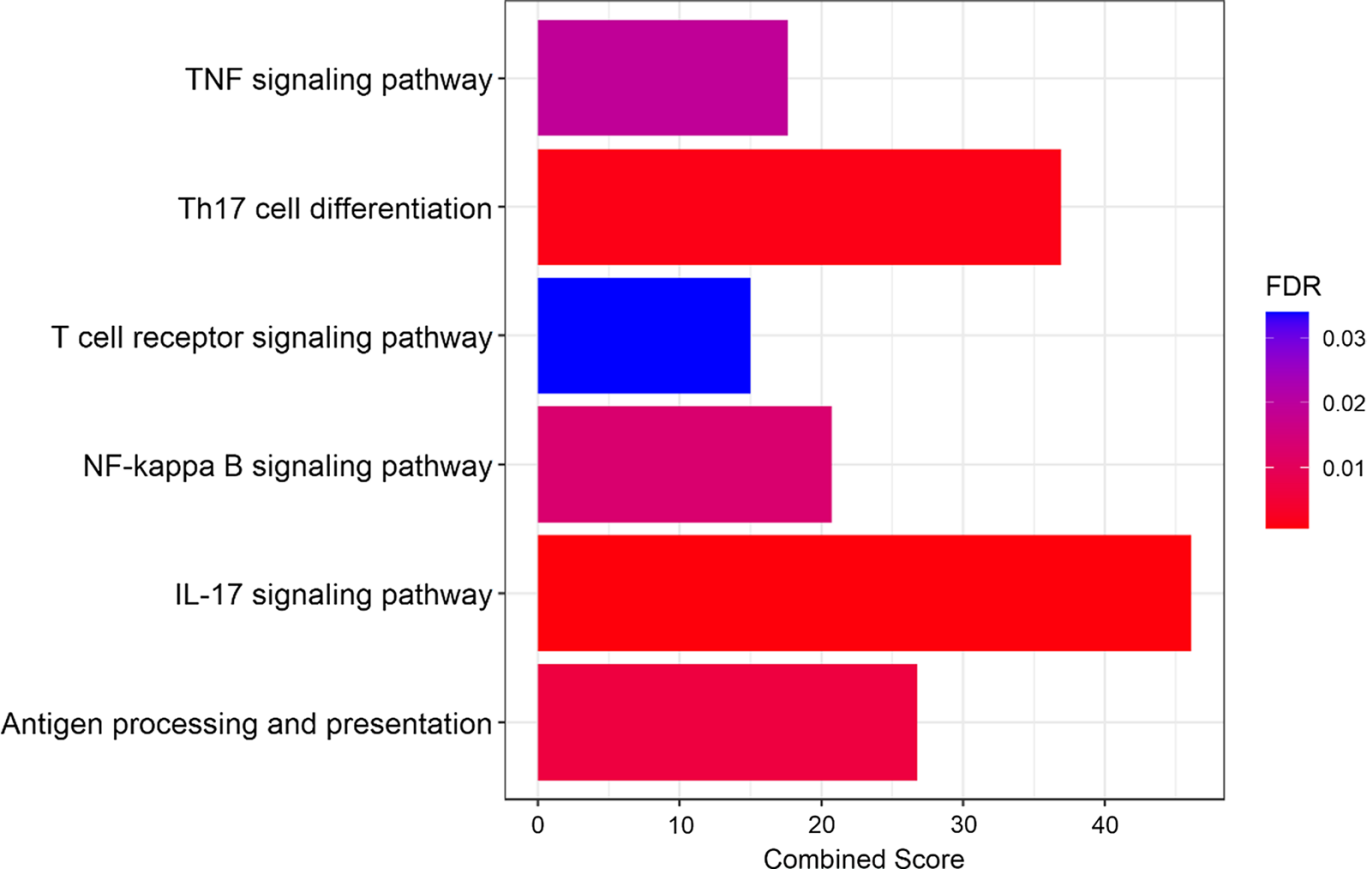
Enrichr pathway analysis showing upregulated eTh17 cell pathways in dendritic cells (characterized in Fig. 3) from lungs of mice exposed to PM compared to vehicle exposed. Significance was tested by Wilcoxon rank sum test with Bonferroni correction FDR < 0.05. Combined score is a combination of the p-value and z-score (calculated by using a modification to Fisher’s exact test) calculated by multiplying the two scores

Studies of allergic asthma in mice have demonstrated that *Il-17*-induced airway neutrophilic asthma was dependent upon *Tnf* and *NF-κB* induction [23, 54, 60, 63]. Looking at the dendritic cell cluster from Figs. 2 and 3, we see five pathways that were significantly (FDR < 0.05) upregulated with respect to eTh17 differentiation and subsequent neutrophilic asthma. Through Enrichr and KEGG, we see increases in Th17 cell differentiation, *Il-17* signaling, *Tnf* signaling, *NF-κB* signaling, and antigen processing and presentation (Fig. 5). This correlates with other published data and demonstrates that the Th17 response is, in part, initiated from cytokine production in dendritic cells. Therefore, this shows a potential mechanism by which dendritic cells promote an Th17 cell response through *Il-17*, *Tnf-α*, and *NFκB* up-regulation, which synergistically activates eTh17 cells.

### ScRNA-seq identifies several distinct T cell clusters

**Fig. 6:**
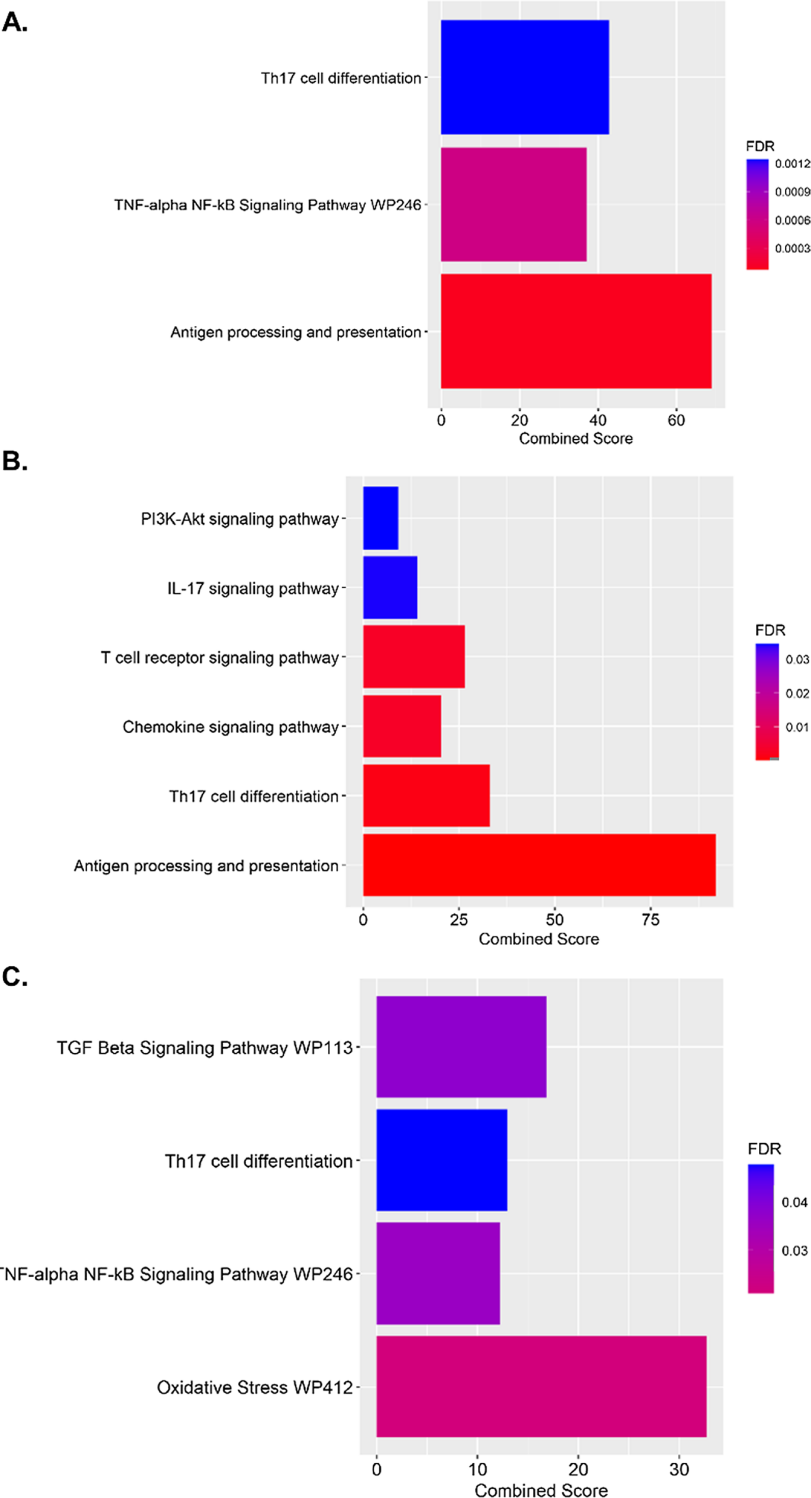
Distinct T-cell populations (characterized in Fig. 3) show dysregulation across immunological pathways essential to eTh17 cell cytokine production. **A** Upregulated Th17 specific pathways associated with PM exposure in alpha–beta T cells. **B** Upregulated Th17 specific pathways associated with PM exposure in NK T cells. **C** Upregulated Th17 specific pathways associated with PM exposure in gamma–delta T cells. Significance was tested by Wilcoxon rank sum test with Bonferroni correction FDR < 0.05. Combined score is a combination of the p-value and z-score (calculated by using a modification to Fisher’s exact test) calculated by multiplying the two scores

**Fig. 7:**
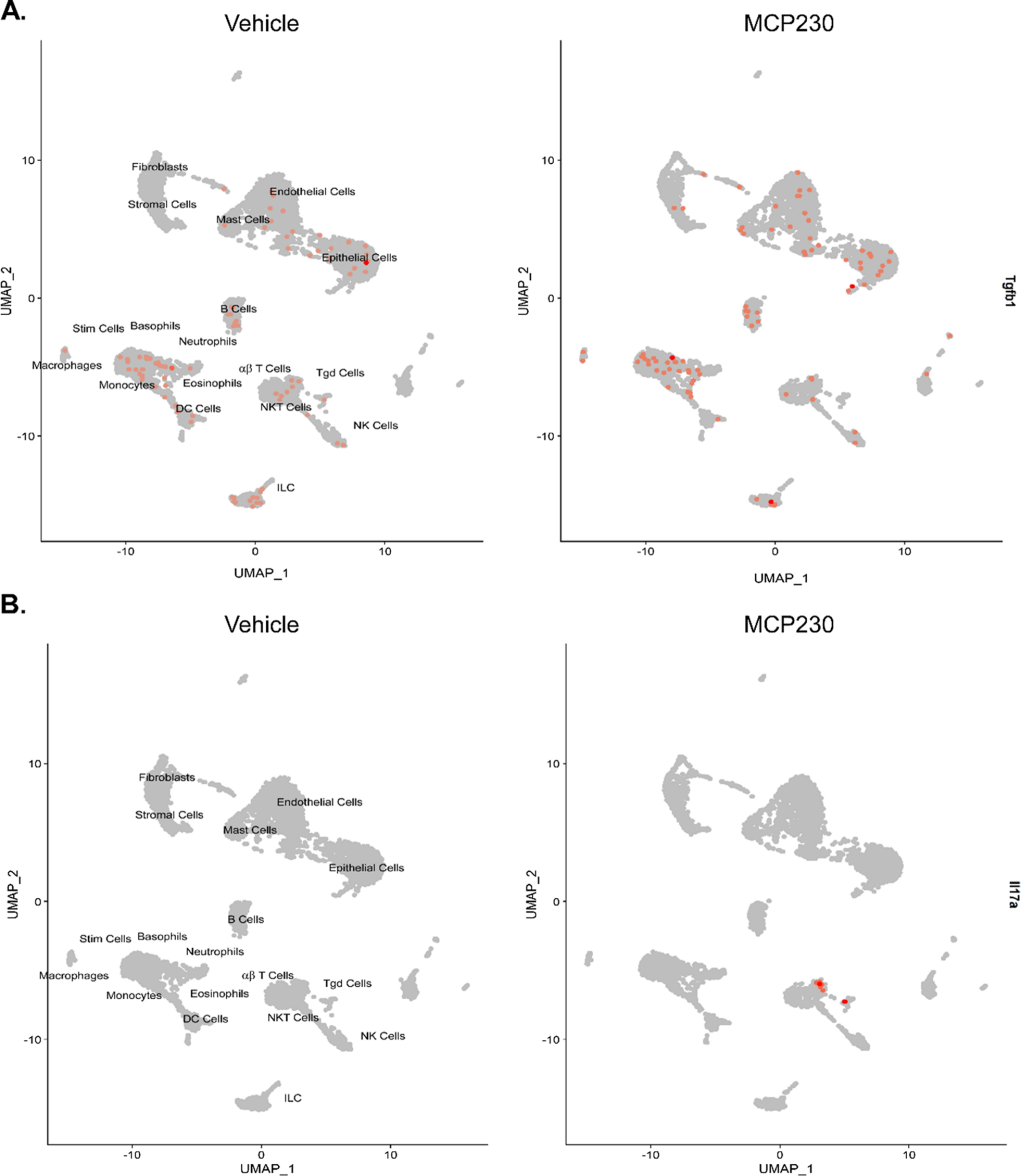
Gene expression of cytokines that induce eTh17 cells in all cells from Vehicle exposed and PM exposed mice. **A** Upregulated Transforming growth factor beta (Tgfb1) was associated with PM exposure in epithelial and endothelial cells. **B** Upregulated Interleukin 17 (IL17a) was associated with PM exposure in alpha–beta T cells. RNA expression levels (colored dots; scale bar on the right) are mapped onto the cells in which they are expressed. Significance was tested by Wilcoxon rank sum test with Bonferroni correction FDR < 0.05

**Fig. 8:**
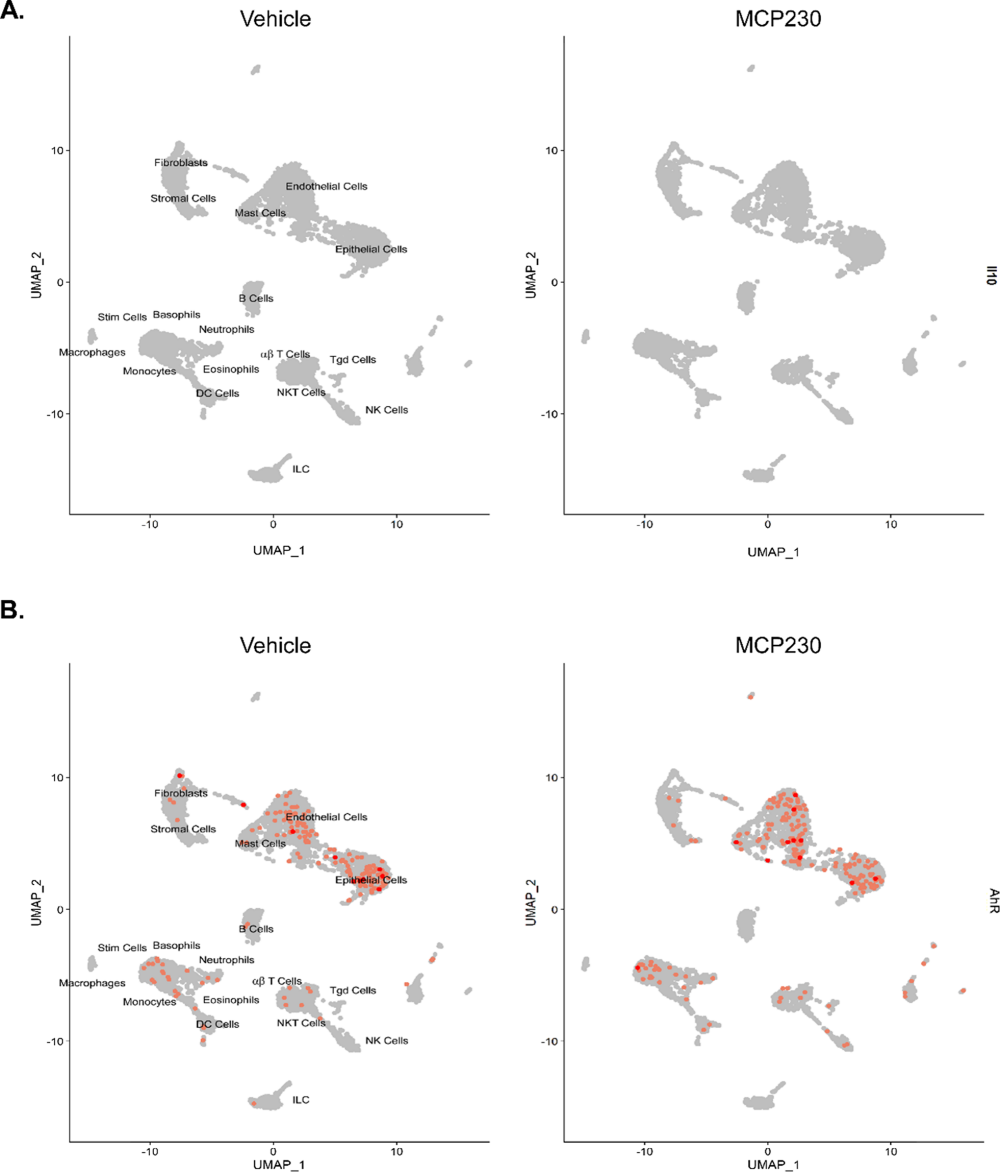
Gene expression of IL10 and *Ahr* in all cells **A** IL-10 wasn’t expressed in any lung cell demonstrating a lack of rTh17 differentiation. **B** No significant changes in *Ahr* expression in PM exposure. Significance was tested by wilcoxon rank sum test with Bonferroni correction FDR < 0.05

Unsupervised clustering using the UMAP protocol found three distinct T-cell clusters, namely, gamma–delta, NK, and alpha–beta T cells. Using the differential expression profile between vehicle and PM exposure, Enrichr identified pathways specific to Th17 differentiation and pro-inflammatory pathways associated with pulmonary eTh17 responses. In all three T cell populations, we observed an upregulation in Th17 cell differentiation (Fig. 6). Alpha–beta and gamma–delta T cells demonstrated upregulations in the *Tnf-alpha NF-κB* pathway, which, as previously mentioned, synergistically activates eTh17 cells. In addition, *Il-17* signaling, *PI3K–Akt* signaling, and *Tgf-β* pathways were upregulated in NK T cells and gamma delta T cells, respectively. As *PI3K–Akt* and *Tgf-β* signaling are essential the induction of eTh17 cells, we can see specifically that the alpha–beta cells are already producing *Il-17a* while epithelial cells, neutrophils, and monocytes produce *Tgf-β* further demonstrating the induction of eTh17 cells (Fig. 7a, b). Meanwhile, across all cells we see no induction of Il-10 demonstrating a distinct lack of rTh17 cells (Fig. 8a). Furthermore, we can see in our cellular map that *Ahr* expression shows no significant change with PM exposure further suggesting that the *Ahr* activation previously seen may not be due to increased *Ahr* expression (Fig. 8b) but rather *Ahr* activation through an alternative pathway as we show in Fig. 4.

## Discussion

Several epidemiological studies have demonstrated the deleterious effects of short-term exposure to combustion derived PM. Although the association of PM and respiratory illness is well established, the underlying mechanisms are not fully understood. Some proposed mechanisms by which PM elicits immune responses have been demonstrated, but missing links as to the specific trajectory that PM follows to trigger pulmonary damage remain. For example, it is well known that PM dictates human airway epithelial cells to express inflammatory cytokines through the *NF*-*κB* pathway [13, 55, 56, 65]. In addition, PM has been shown to increase oxidative stress through the activation of inflammatory cells along with being able to directly generate ROS from the surface of PM [29, 68]. In this study, we demonstrate the effects that elevated levels of PM exposure has on lung cells during innate immune response and the specific pathways that PM alters during innate exposure.

First, we utilized the mouse drug metabolism profiler of the RT2 PCR Qiagen arrays to show the activation of genes controlled by *Ahr* in response to short-term exposure of PM, which showed upregulation of tryptophan metabolism, steroid hormone, biosynthesis, retinol metabolism, glutathione metabolism, and thyroid hormone synthesis. This upregulation suggests that exposure to PM elicits an antioxidant response and increases oxidative stress. Since *Cyp1a1* was highly upregulated in our RT2 profiler array, we looked at *Ahr*, the transcription factor that drives *Cyp1a1* expression, and found in our single-cell analysis that epithelial cells had multiple genes upregulated in the *Ahr* wikipathway, namely, *Hsp90aa1, Jund, Ptges3, Cdc37, Nfe2l2, Gclc, Cd36, Tnf,* and *Egfr1*. This is interesting, as it shows a potential alternative pathway to *Cyp1a1* expression to PM acting as a ligand, as we previously thought [11, 27, 44]. *Ahr* activation has recently been studied for its role in Th17 activation and consequently in hypercytokinemia, a severe immune reaction in which the body releases too many cytokines, as an early host response to respiratory infections. Understanding this *Ahr* activation is essential to elucidating the specific downstream effects prompted by PM exposure.

Since eTh17 cell differentiation is dependent upon *Tnf*, *TGF-β*, *Il-6*, and *Il-8* induction, we looked at our single cell analysis to trace what cells are involved in the PM induced neutrophilic asthma. In epithelial cells (Fig. 4), we show distinct changes affecting a multiplicity of molecular pathways including *Il-17* signaling, *Ahr* activation, and *Egfr* signaling pathways. Epithelial cells are the first cells to encounter foreign objects such as PM in the lungs. So, the activation of specific pathways in epithelial cells will inform further responses in the immune system through cytokine initiation. Previous studies have established that PM promotes bacterial invasion of airway epithelial cells by attenuating ROS, destroying tight junctions, and causing epithelial to mesenchymal transitions [12, 50, 59]. In this paper, we present data suggesting that PM induced epithelial transcriptomic changes are responsible for the activation of *Ahr* through *Ahr/Arnt* activation/nuclear translocation and an increase in *Egfr* expression, ultimately resulting in neutrophilic asthma as we have previously shown [27]. *Ahr* ligand activation leads to the production of xenobiotic response elements such as *Cyp1a1*, *Cyp1a2*, and *Cyp1b1*, however, the ligand activation of *Ahr* also releases the non-receptor tyrosine kinase c-src in the cytoplasm. C-src kinase translocates to the cell membrane where it activates *Egfr* as we see in Fig. 4b [9, 16]. *Egfr* activation further leads to *Erk*1/2 pathway activation promoting the transcription of *cox-2* which has been shown to be essential in eTh17 differentiation [17, 37]. This suggests that there could be new potential therapeutic targets for neutrophilic asthma treatment in the form of blocking *Egfr* or the activation of c-src.

Following the upregulation of *Ahr* specific cytokines and Th17 specific cytokines in epithelial cells we see that dendritic cells further produce cytokines involved in Th17 cell differentiation, *Il-17* signaling, *Tnf* signaling, *NF-κB* signaling, and antigen processing and presentation pathways. Neutrophilic infiltration is dependent on *Il-17* signaling through *Il-17* induced proinflammatory cytokines and chemokines in lung structural cells promoting neutrophilic infiltration [66]. Both *Il-17* and *Tnf* have been shown to induce *Il-6* and *Il-8* which are essential to eTh17 cell differentiation. With anti-*Tnf* therapy in severe asthmatic patients there was a decrease in sputum neutrophil levels, but not pulmonary neutrophilia [3, 7, 25, 51]. Thus, the eTh17-*Tnf* axis may be involved in the development of neutrophilic asthma.

There were other significant differences in the expression profiles between the three distinct T-cell clusters found in our single-cell RNA sequencing analysis. We found αβ and γδ T cells demonstrate upregulations in the *Tnf-α, NF-κB* and Th17 differentiation pathways. In addition to Th17 differentiation, we further found upregulation of *Il-17* signaling in NKT cells, while seeing upregulation of *PI3K–Akt* and *Tgf-β* signaling in γδ T cells. *PI3K–Akt* can induce *IL-6* and *IL-8* and likewise *Tgf-β* have been shown to increase Th17-associated cytokine secretion and T-cell differentiation toward Th17 cells [8]. NKT cells have the ability to produce *Il-10, Il-13, Ifn-ɣ,* and *Tgf-β* [48]. *Il-10* is vital in the differentiation of rTh17 cells while *Il-13, Ifn-ɣ,* and *Tgf-β* are pro-inflammatory cytokines that further stimulates eTh17 cell differentiation. This signifies how early lymphocytes such as NKT, γδ T, and αβ T cells inform the immune response later and can promote neutrophilic asthma [35, 42].

Th17 cells have been known to play both protective and pathogenic roles in various diseases [6, 18, 26, 43, 52, 58]. Therefore, future research would be to focus on the transcriptomic changes at different times throughout exposure to fully map the immune response and cellular composition changes associated with PM exposure.

Finally, it is important to point out that although this data is in line (i.e., Th17 responses following exposure to EPFR containing PM) with our previously published works, there are limitations to this study. First, this is an analysis of complex biological datasets at a static time point in response to an injurious event in vivo. Second, enzymatic digestions used to isolate these cells may modify the transcriptome affecting the resulting data. Third, there are many computational steps involved in analyzing such scRNA data including identification/mapping of corresponding cell populations across data sets, normalization and reduction of dimensionality—each with its own assumptions. Fourth, this work was done on a relatively small number of samples due to cost and only looked at poly A RNA excluding non-coding RNA.

## Conclusions

Our study shows the diverse and distinct transcriptome changes of epithelial, dendritic, and T-cells brought on by exposure to elevated levels of PM. Our data unveils potential pathways and gene expressions explaining the phenomena of neutrophilic asthma that we and others have previously observed following PM exposure in mice and humans [35, 42, 60]. While other studies have shown an innate immune response to PM exposure through microarray data, our study is the first to investigate the innate immune response on a cellular level through single-cell RNA sequencing.

## Supplementary Information

**Additional file 1: Figure S1.** Elbow plot and jackstraw plot of principle components to determine the optimal number of PCs to construct the UMAP plot

## Data Availability

The datasets generated and/or analyzed during the current study are available in the SRA Accession database: PRJNA666321.

## Abbreviations

PM: Particulate matter
EPFR: Environmentally persistent free radicals
Ahr: Aryl hydrocarbon receptor
ScRNA: Single cell RNA sequencing
Th17: T helper cell subset 17
eTh17: Pathogenic T helper 17 cells
rTh17: Regulatory T helper 17 cells
ROS: Reactive oxygen species
COPD: Chronic obstructive pulmonary disease
OA: Oropharyngeal aspiration
UMAP: Uniform Manifold Approximation and Projection
NK: Natural killer cells
NKT: Natural killer T cells
