# Supplementary figures and images for: Association between particulate matter containing EPFRs and neutrophilic asthma through AhR and Th17
Source: Respir Res. 2021 Oct 26;22:275. doi: 10.1186/s12931-021-01867-w (PMC8549224; doi:10.1186/s12931-021-01867-w)

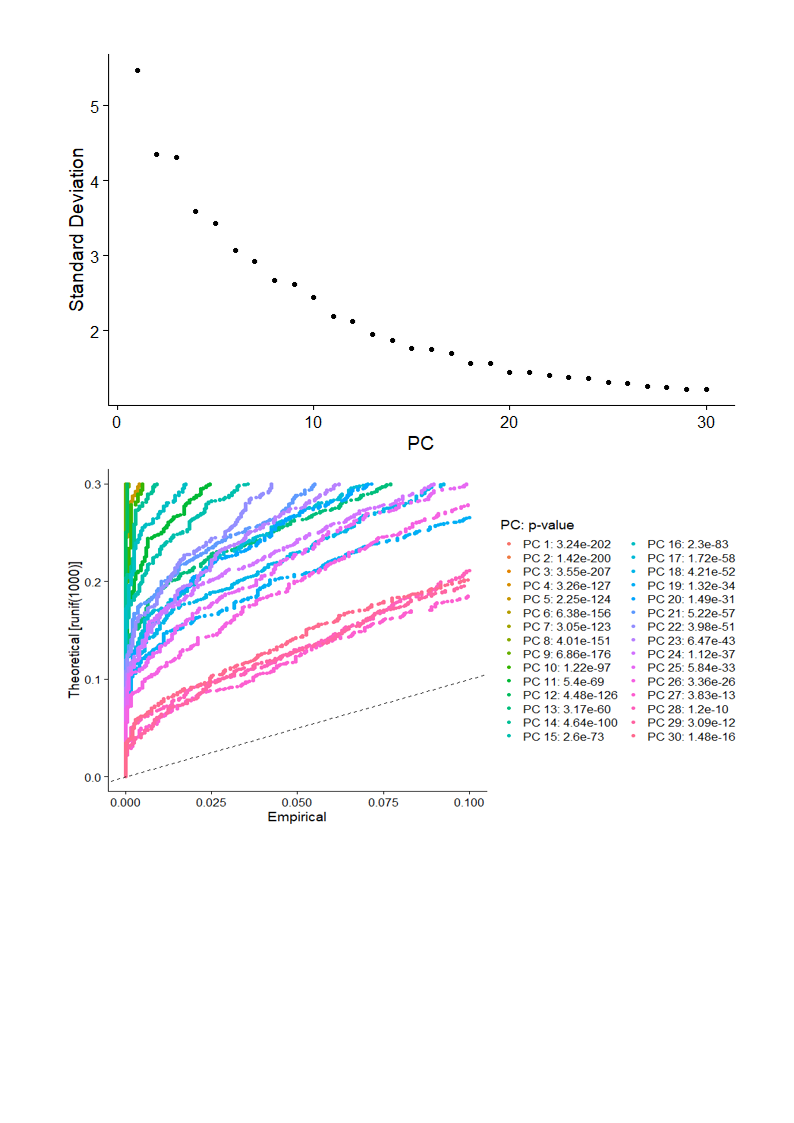

Supplement: Supplementary file 1 — Additional file 1: Figure S1. Elbow plot and jackstraw plot of principle components to determine the optimal number of PCs to construct the UMAP plot [file 12931_2021_1867_MOESM1_ESM.png]
